# Porrigo, or Tinea Capitis

**Published:** 1821-12-01

**Authors:** 


I. *A Practical Essay on Ringworm of the Scalp, Scalled Head, and the other Species of Porrigo.* By Samuel Plumbe, Member of the Royal College of Surgeons of London; of the Medical and Chirurgical Society, &c. One vol. 8vo, pp. 104, with two coloured plates. London, 1821.

II. *On Tinea.* By M. Alibert, Diction. des Sciences Medicales, vol. 54, pp. 48, octavo. Paris, 1821.

Much has been written on tinea capitis, or porrigo—and especially on that form denominated porrigo scutulata, ringworm of the scalp, scald head, and teigne granulée; but that little has been effected in respect to a knowledge of the nature and treatment of this disgusting disease, we have (besides the evidence of our own senses) the authorities of Alibert and Bateman—the former the greatest dermologist of the present day. "Que trouve-t-on," says Alibert, "dans les auteurs touchant la nature et le caractère specifique des *teignes*?—Des renseignemens incertains, des dissertations vaines, des details vagues." Bateman says that this unmanageable form of porrigo often continues for several years. "Whether the circles remain red, smooth, and shining, or become dry and scurfy, the prospect of a cure is still distant—for the pustules will return, and the ulceration and scabbing will be repeated."

Most of the forms of porrigo being contagious, the disease is propagated, by means of schools, to a great extent, even among the higher classes of society; and consequently it becomes a subject of no trifling interest to the practitioner, who, being often foiled in the treatment, has sometimes the mortification of seeing the patient placed under the care of another, and not unfrequently put into the hands of a Charlatan. On this account, we shall be excused in going farther into the subject than is usual in Journals or Reviews, especially as some of the sources, whence we shall draw our information, are inaccessible to the great majority of our readers.

Mr. Plumbe, who has lately directed much attention to the disease under consideration, introduces, in the first chapter of this work, some ingenious remarks, anatomical and physiological, on the hair and integuments which it covers. It has been ascertained that the hairs have their origins completely beneath the under surface of the cutis of the scalp. The layer of adipose membrane *there* appears partially interwoven with the inner surface of the integument, and firmly attached also to the bulbs of the hair, which seem to be implanted therein. The hair then would seem to be independent of the cutis, as far as regards its nourishment—the cutis, a vascular and highly sensible structure, being merely penetrated by the hair, which appears to draw its support, and also the oleaginous secretion covering it, from the adipose structure beneath.

"The fact that the scalp is pierced by the hair and has little or no share in its production or nourishment, I am particularly desirous of impressing upon the attention of my readers. Reasoning from analogy we should be justified by this consideration only, in concluding that the latter *may* possess when the former is in a state of disease all the properties of extraneous substances. As regards the common ring-worm of this part, it will be uniformly found evincing these characteristics in the mildest as well as most severe forms of the disease." 16.

Mr. Plumbe, in his 2d chapter, commences with *porrigo sculutala,* omitting altogether the porrigo larvalis, or crusta lactea, as not naturally belonging to this class of diseases, either as to character or cause.

1. *Symptomatology.* According to Mr. Plumbe, the falling off of the hair is usually the first symptom which discovers the disease. When the scalp is examined, under such circumstances, it exhibits a somewhat scurfy and slightly reddened appearance. The remaining hair of the diseased part is thin, and irregularly scattered over it; the greater portion appearing to have been broken short off near the scalp, their roots still retaining their situation. Those which remain generally drop off if friction be applied. These are the primary appearances, the achores, or minute straw-coloured pustules, not being necessary, Mr. P. thinks, to constitute the disease, as they are not seen till later, when some degree of itching and irritation of the part has been felt.

"Though the achores mentioned by different authors who have preceded me as being the most important feature of this disease, are not seen at its first commencement, they are usually soon making their appearance after the hair begins to fall off. The itching and irritation commencing at the same time, the child who is the subject of it soon ruptures a few of them, and spreading by the frequently repeated application of the nails to the spot, their contents cover the adjacent parts of the scalp, extends the disease with great rapidity upon it; the same destruction of the hair and subsequent pustulation marking its progress.

"When pustules are noticed they are uniformly found with hairs growing through them; and if the disease has existed for a considerable length of time and destroyed the greater part of the hair of the part, such pustules are found proportionately reduced in number; but still surrounding the few straggling hairs which remain: each single minute pustule appearing to be dependant on the hair in its centre.

"If the hair, as sometimes happens, be completely eradicated from the spot where the disease first appears, the skin assumes an apparently healthy character: the disease, as regards this particular spot, may be said to have exhausted itself." 23.

That the disease when once formed on the spot spreads only to a small extent, by the application of the infectious matter, (and not from the mere communication of the specific action of the vessels of the part to those adjoining,) is,

Mr. Plumbe thinks, an important fact in the pathology and treatment, since, if it be admitted, (and he thinks no doubt will be entertained by those who attend minutely to the progress of even a single case,) it will be obvious that the present routine management, as regards applications to the part, is inefficient or futile, since it is not to be expected that they can neutralize or decompose the specific infection secreted from the parts. "The common attentions of a cleanly nurse are infinitely more effectual, and that for obvious reasons, in this hitherto terrible complaint, than any medicinal application, whatever, where cleanliness, is neglected."

The assertion of Dr. Willan, that all the different species of porrigo may be produced from the same contagion, is questioned by our author, as not according with his observations.

"Not only have the results of the common accidental occurrence of the different species of the disease afforded proofs of the contrary, but experiments instituted for the purpose have invariably supported the opposite conclusion." 27.

The porrigo larvalis, or crusta lactea, is acknowledged by Dr. Bateman to be non-contagious, and so far as our author's experiments have gone, it appears probable that the p. scutulata, furfurosa, and lupinosa *only*, are the results of one specific contagion, the points in which these differ from each other being of very small importance. The porrigo decalvans is of such rare occurrence as to render it difficult to form a decided opinion respecting the mode of its production.

The nature of p. favosa is better ascertained. "In no case," says Mr. Plumbe, "does this form of the disease appear by infection, to produce either of the other species. Whether the matter secreted be inserted under the cuticle or simply applied by friction, the same favous pustule is produced as the original." It is almost needless to remark that ring-worm of the scalp, the disease now under consideration, is very different from what goes by the same appellation on other parts of the body. This last rarely shews pustules, and never vesicles or tetters; but is usually marked by a lightish redness of the spot, and slight exfoliation of the cuticle. "The centre of the patch appears to have lost the greater part of this covering, while a morbid accumulation of it marks the extension and line of margin of the disease." The ung. hyd. nit. diluted with the cerat. superacet. plumb. we have found the most effectual remedy in this last cutaneous affection, assisted by frequent ablution with soap and water.

The statements handed down to us of the effects of p. scutulata, &c. altering the colour and strength of the hair, Mr. Plumbe thinks, are contradicted by daily experience, as, whatever may be the effect produced on the hair growing on the part at the time, the structure secreting the hair is not affected by it, and when the disease is subdued, the hair will grow strong and healthy as ever, especially after being once or twice shaved. When extensive ulceration of the scalp has been produced by mismanagement or neglect, the case is materially altered. Then the production of new hair will, of course, depend on the degree of mischief which the parts secreting it may have sustained. Mr. Plumbe considers the porrigo scutulata, of course, as a *local* disease, quite independent of constitutional affection.

Without advocating such barbarous modes of practice as the pitch-cap, formerly in use, Mr. Plumbe thinks the said practice was founded in a just principle.

> "The removal of the hair by the roots, where the pustules of the disease has loosened them, is a method absolutely necessary to the speedily checking it; but it is obvious enough that the forcibly drawing off the pitch cap, must equally draw up the roots of the sound hair to which it is attached on the adjacent parts." 41.

The separation of the hair round which any pustule is discovered, is productive of little or no pain, and is readily accomplished by a pair of small forceps.

Mr. Plumbe observes that the term "scalled head" has been rather too indiscriminately applied to porrigo favosa and different stages of p. scutulata, though a distinction between these last is highly necessary for the purpose of successful treatment. Mr. Plumbe suggests the restriction of "scalled head" to the advanced stages of p. scutulata.

> "Notwithstanding the absence of that copious exudation of fluid even in the most advanced stages of the scutulata, which marks the favous species, and the existence of many other very important differences in the two diseases; the same methods of treatment are still inculcated by the majority of those who have preceded me, as applicable to both. If medical men, taking on themselves the characters of authors, will continue to propagate ideas unsupported by actual attentive observation of the diseases on which they write, as guides for their professional brethren; (and it is to be lamented that this has been much too often the case) confusion and mischief must necessarily arise: and in this particular case, a practical man would have no hesitation in denouncing an author of this description, as a most mischievous member of society." 46.

II. *Treatment of P. Scutulata.* In this important part of the subject our author conceives that he can derive little assistance from those who have preceded him. Considering the discharge from the numerous minute pustules, (continually forming,) as capable of producing or extending the disease to sound parts in the neighbourhood, Mr. Plumbe's preliminary step is to effect the evacuations of as many of the pustules as possible, by pinching up the skin between the finger and thumb, and carefully washing away what is thus forced out. Previously, however, to this operation, all hairs are to be plucked out, *which come away readily and without pain,* a precaution which he deems necessary whether pustules have formed in great numbers or not. Shaving the part, though proper in all cases, after the loose hairs have been removed, is no effectual substitute, Mr. Plumbe thinks, for the extraction alluded to. The loose hairs removed, and the pustules evacuated,

"Some astringent application, possessing *the power of taking from the secretion its infectious properties*; *and, at the same time, sufficiently powerful to constringe the vessels from which it flows and lessen its quantity*, may be made use of.

"A solution of the sulphate of copper has been employed in some cases for this purpose. I believe the object to be more completely accomplished however, by rubbing this preparation in a finely powdered state on the part, and then washing it off." 63.

The extraction and shaving of the hair usually produces some inflammation and production of a few more pustules, unless some sedative applications are used for a day or two. A careful examination should be instituted every morning, and if any pustules appear, they should be removed at once by the sulphate of copper, as at first applied.

"After two or three repetitions of this application, no fresh appearance of pustules take place, and the circle of the disease is marked by small thin scabs of a darkish colour, and the same characteristics in other respects as the common exudation from abraded surfaces of the cutis. These scabs separate in a few days, bringing with them a few of the remaining hairs which have separated, and leaving a shining red and irregular surface, which gradually loses its inflammatory character, having now and then a little scurf forming on it till the new hair begins to appear." 64.

Sometimes it happens that, when the new hair is quickly reproduced, after the disease has been subdued, the excitement it occasions is followed by a slight fluid secretion concreting into minute scabs, retarding the production of a strong and healthy cuticle. Where numerous pustules have been formed, we need hardly expect a strong and healthy crop of hair in less than six weeks or two months:—at the end of three months it will usually have attained its original strength.

Mr. Plumbe animadverts on the assertion of Willan, that extraction of the hair does more mischief to the scalp in one day, than the disease, if left to itself, would effect in three years, where cleanliness is observed. He thinks this cannot be true, since "the organic structure producing the hair is neither drawn out with it, or sustains any mischief by the violence employed."

"With due deference to the author in question, I should answer this interrogatory by asserting that the disease itself, *may*, under mismanagement and neglect, become a means (by subsequent ulceration) of inflicting a permanent and irreparable injury to the scalp: but that the forcibly drawing out of *every hair*, sound or unsound, is not adequate to the production of any effect on the part beyond a temporary privation of this necessary covering." 69.

At the same time our author deprecates the idea of forcibly depilating the scalp by tearing off the pitch cap, dragging with it the sound and unsound hair, together with the scabby secretions of the tender and irritated surface—"inflicting as much pain as the most resolute of dispositions can support." "From all these inconveniences the use of a pair of forceps is perfectly free;" as they are applied only where the disease exists, and to hair already loosened, the slightest degree of force being sufficient to remove them.

Mr. Plumbe observes that a considerable period must necessarily elapse between the cessation of growth, and the entire separation of the hair, during which time the hair itself may be considered an extraneous body exciting irritation and keeping up the disease. "Let it not be said, therefore, that no necessity exists, under these circumstances, for its removal."

It is evident that the treatment, above described is only applicable to a state of simple ring-worm of the scalp, and requires modification where the case is of long standing, and where accumulation of scabs, portions of ulcerated surface, and high irritation of the vessels, exist. The following passage will convey to the reader some idea of Mr. Plumbe's practice in these obstinate cases.

"In a case of this kind the subject of the plate No. 1, and in several others of a similar description; the difficulties of subduing the excessive irritation of the disease were for a considerable period considered insurmountable. Fomentations, poultices, and cold lotions, were successively and diligently applied; each for a sufficient length of time to have produced, under common circumstances, an effectual check to inflammatory action. Still the redness and heat of the part remained obstinate, and where a few straggling hairs were seen, a constant production of new pustules were discovered, as fast as others were removed. A total extirpation of the remaining hair over the whole surface was eventually accomplished; the ulcerated portions healed; and the fluid secretion diminished; the inflammatory redness and heat of the part continued however; and around the healthy margin, new pustules and scabs affecting the sound hair were every day appearing.

"The preceding applications, with others of various descriptions, were changed one for another without success, till a small spot, whence the hair had been first removed, was occupied by fresh; and immediately after pustules appeared among it. It appeared now, that any further attempts to get rid of the disease would be frustrated by the increased irritation of the new hair, which might be speedily expected to spring up over the greater part of the surface: but except on the spot I have mentioned, no more hair appeared; and I was led, shortly after, to endeavour to apply pressure by means of adhesive straps and bandages, with the cold lotion in conjunction. By the diligent application of these for two or three weeks, a material change was produced; and the scalp began to assume an appearance more nearly approaching to health. To completely subdue the diseased action was a work of much time; but I had the satisfaction of seeing eventually, (as I have already stated in a previous allusion to this case) the part completely covered with long and glossy hair." 74.

In other cases of similar character, and of four and six years standing, Mr. Plumbe has experienced proportionably less obstinacy; "but in no single case has the plan last mentioned (pressure and cold applications combined) failed in subduing the disease." In the majority of these cases, the extraction of the hair was necessary, in consequence of the obstinate repetition of the pustules, where any hair appeared on the diseased surface.

The foregoing description of the local treatment comprehends every thing on which our author is able to speak with decision, and from personal experience. The application of greasy substances not having the power (and none have) of destroying the infectious properties of the pustular discharge, Mr. Plumbe cannot approve, for he has frequently seen the disease spread with greater activity, after such applications.

Mr. Plumbe cannot agree with Mons. Alibert, in affixing a constitutional origin to all kinds of tinea. He thinks Mons. A's remarks ought to have been almost exclusively applied to the porrigo favosa. At the same time, he admits that, in bad cases, the constitution sympathises, and a feverish state of the system is rarely absent. Here constitutional treatment is necessary of course.

3. *Porrigo Favosa.* Willan has accurately described this disease. The very large quantities of secretion poured out from the diseased surface and forming the scabs, distinguish it from all others. The achores or minute pustules of the P. Scutulata and the favi of the present subject, have hitherto been considered as varieties of the same genus, differing chiefly in magnitude; but the propriety of this our author questions. The achores, he observes, are deeply seated in the cutis, and obviously occasioned by the irritation of the hair. The favi, on the other hand, occur on every part of the body, and are, in most cases, formed by the mere effusion of fluid under the cuticle from the vessels on the surface of the cutis, the distended cuticle forming the parietes of the pustule, and being the only means of retaining its contents in its place, ulceration of the cutis not occurring. The matter of one is deeply imbedded in the structure of the cutis—while that of the other lies on its surface.

"They are erroneously supposed to be the produce of the same infection, but the application of the matter of each only excites its own disease. In one the hair falls off; in the other it is very little affected, nor is its removal, except by shaving, necessary to the cure." 80.

Porrigo scutulata is considered by our author and others, as a merely local disease, not connected with any particular state of constitution. But the *favosa* arises (spontaneously) in robust and apparently healthy constitutions *only*, and may usually, Mr. Plumbe thinks, be referred to constitutional disorder arising from improper food and want of exercise. "It may be perhaps not unsatisfactorily looked upon as the result of an effort of the constitution to get rid of inconveniences arising from the above state." This view of the subject will be enlarged when we come to M. Alibert's observations.

"In removing the scabs of this disease, whether existing on the head, or other part; we discover a reddened and inflamed surface, pouring out with excessive rapidity a viscous transparent fluid, which speedily dries and forms fresh scabs of various shades of colour, from a transparent yellow, to dark brown. An areola of inflammatory redness, usually surrounds the part, as if the whole energies of the vessels of the diseased spot, and adjacent cutis were called forth in keeping up the fluid secretion." 86.

In the treatment of this disease the general constitution must be acted on, and it requires more frequently depletion and alteratives than tonics, which have been recommended. Applications which allay irritation and relieve pain, are useful auxiliaries. When, however, the disease is situated on the scalp, some modification of treatment is necessary. Here there is constant irritation from the hair, while the glutinous secretion lodging prevents any application to the diseased surface. In protracted cases, where the disease is aggravated by myriads of vermin and accumulated secretions, the removal of filth, even at the risk of some pain, must be attempted. The scabs must be soaked in warm water and soap, and the hair removed by the razor, as preliminary steps. Whether ulceration exist or not, fomentations and poultices are necessary to subdue the inflammatory state of the parts; after which, attention to the general health is sometimes all that is necessary.

When the disease has existed long, however, it will require more active external remedies. An effectual application, Mr. Plumbe observes, will be found in a solution of caustic or sulphate of copper (argent. nit. ℈j. aq. distillat. ℥j. or cupri sulph. ʒj. aq. ferv. ℥ss.) in such cases. These fluids are to be applied, by means of a camel's hair brush, to the abraded surface twice or thrice a day until the discharge ceases.

The *p. favosa,* like ring-worm, spreads rapidly by infection. When a favous pustule is thus produced by inoculation, it may often be checked speedily in its course. Its contents may be removed at once, and if the abrasion is of sufficient consequence to require a poultice, it may be applied. If this cannot be done, frequent ablution with warm water will diminish the irritation, remove the secretion, and prevent extension of the disease.

4. *Porrigo Furfurans et Lupinosa.* Our limits will not permit us to analyse Mr. Plumbe's fifth and sixth chapters on *P. Furfurans,* and *P. Lupinosa.* The *former* of these, being, like p. favosa, generally owing to constitutional disease, requires the same treatment, local and general, as the p. favosa, and sometimes as the p. scutulata. The *porrigo lupinosa,* Mr. Plumbe considers to differ so little from common ring-worm of the scalp, as to render it doubtful whether it ought to receive a distinctive appellation.

Mr. Plumbe concludes his work by pressing on the attention of practitioners the paramount necessity of cleanliness in all species of porrigo.

We have, we hope, exhibited sufficient specimens of the matter of this work, to induce our readers to seek a more intimate acquaintance with the contents of the original. The style is plain and generally perspicuous, with the exception of a considerable defect in punctuation—the semicolon being far too often used, where a comma only was necessary. This, however, is a *point* of no great consequence in a practical work on a subject which certainly neither requires, nor admits of, much literary embellishment.

It now becomes our duty to notice the opinions and practices of an illustrious foreigner, M. Alibert, whose opportunities for observing cutaneous diseases, at the HOPITAL ST. LOUIS, are certainly (and we pay no great compliment to the propreté of our continental neighbours by saying so) unequalled in any capital of Europe—and particularly in this capital, where more than a million of inhabitants can hardly furnish subjects for a small dispensary, erected lately for the relief of diseases of the skin.

1. *General Phenomena of Tinea.* The individuals affected with tinea, generally feel, at first, a pruritus, more or less violent, on the head. The scalp, on certain points of its surface, next becomes red, chaps, or even becomes a little tumefied. A swelling of the cervical glands sometimes accompanies the complaint—more rarely a head-ache. The itching daily increases; and pustules or vesicles are seen surrounded by an inflamed areola. In some cases no trace of ulceration can be perceived, a reddish viscid humour appearing to exude from the dilated mouths of the glandular follicles. Presently the hair becomes agglutinated by this viscid humour, which issues, flow after flow, resembling melted rosin, forming crust upon crust of scabby or scaly layers, horrible and disgusting to behold! Meantime a putrid sanies beneath corrodes the hairs even to their bulbs, destroys the neighbouring cellular tissue, and threatens the cranium itself. Some of these afflicted fall a prey to violent nocturnal pains—others into a state of emaciation which entirely arrests their growth.

It is more especially when tinea is congenital, or its treatment neglected, that it commits such dreadful ravages. It is then that we see abscesses form in the scalp—glandular swellings in the occiput, neck, shoulders, arm-pits—immense enlargements of the ears—redness, lacrymation, irritation of the eye-lids—disgusting odour from the confluent pustules—falling of the hair—torpor and inaptitude of the intellects—defect of physical power—even of the generative process. Such is the lamentable portrait drawn by Alibert, who had ample means of copying from Nature.

Our author observes that, with the exception of crusta lactea, the different species of tinea rarely attack infants while suckling. The great majority of instances commence after two years of age, and the complaint commits the greatest ravages during the first septenary period. It rarely continues after that. Some species of tinea, however, (the *teigne faveuse,* which appears to be the *porrigo lupinosa* of Willan and Bateman) are more prone to occur after the period of infancy, several examples of which are adduced. It is not with tinea as with some other eruptions. Nature throws it out for purposes, not well known to us at present; and she cures it at or before puberty, even without any assistance from medicine.* Of all the species of tinea, the *teigne faveuse* (porrigo lupinosa) is the most frequent in the Hopital Saint Louis. Tinea mucosa (crusta lactea) abounds in the provincial towns; but as it happens during lactation, mothers rarely send their children to an hospital at such a period.

2. *Etiology.* It is useless, as M. Alibert justly observes, to notice the absurd notions of the ancients respecting the causes of tinea—some placing it to the account of bile, others to an alcali, an acid, an acrid humour, &c. &c.† We must attribute the phenomena of tinea to certain laws of the animal economy implanted there by our creator. We see one species (crusta lactea) disappear when lactation is over—we see other species for the most part disappear spontaneously, when puberty declares itself. Can we hesitate, then, to consider these exanthemata as connected with the laws and operations of the constitution?

Here our author relates a curious case of chronic enteritis, where the child was daily wasting with fever and abdominal irritation. The physician, M. l'Homme, inoculated the forehead of the patient with matter from tinea mucosa (crusta lactea) and soon produced the disease. In ten days the the whole face was in a mask of humid scabs, and from that period the abdominal pain and sensibility began to decline.

---

* This is a fact which militates most decidedly against tinea being a perfectly local disease.

† M. Alibert makes a keen remark at the beginning of his paper, which is worth recording. "When I see," says he, "the disease every day before me, why should I run to the Greeks and Arabs for a description of it?" If Willan and Bateman had taken this hint, what a huge mass of useless learning would have been spared? "Tout étalage d'erudition ne serait q'un vain jeu de l'esprit, sans avantage pour la science." Alibert, *in loco citato.*

The child was soon restored to health. M. Alibert speaks of great advantages resulting from this species of inoculation, under his own observation. The following doctrine may not be believed in this country; yet we are inclined to think that it is not devoid of some foundation in truth.

"All the different species of tinea, however intractable their character, tend to some useful purpose in the animal economy, by determining towards the skin certain noxious principles superabounding in the system. Hence we may account for those fatal accidents that have followed a repulsion of these eruptions, as suppuration of the encephalon, hydrocephalus, enlargement of the mesenteric glands, &c. as witnessed by Forestus, Bonetus, Hoffman, and others."

Nature, however, sometimes opens other outlets, when these morbific humours are suppressed. M. Alibert has seen obstinate diarrhœa succeed the suppression of crusta lactea. A young woman at the St. Louis was affected with tinea furfuracea, to which an ointment was applied, composed of sulphur and hog's lard. As soon as the itching and irritation of the scalp were allayed, a violent pruritus was felt in the pudendum, succeeded by an eruption there. The ointment being laid aside, the irritation of the scalp returned, and the pudendal eruption and pruritus vanished. It was also observed, that, in proportion as the tinea was mitigated by the sulphur, a thick and copious sediment appeared in this young woman's urine.

The records of Saint Louis and M. Alibert's private experience authorize him to assert that a predisposition to tinea is hereditary.

In respect to the contagious character of tinea, M. Alibert is somewhat sceptical. He positively asserts that it is generally very difficult to inoculate a person with the matter of tinea, and that he has seldom succeeded in the attempt. M. Gallot, one of the physicians at Saint Louis, has made many experiments to resolve this question, and the result of his observations is, that the contagious nature of tinea has been greatly exaggerated—and that it is seldom transmitted from one to another, unless there be some constitutional predisposition in the recipient.

3. *Pathology*. The *seat* of tinea has given rise to much discussion among pathologists. Many of these have placed the original seat of the disease in the bulbs of the hair; but there is no foundation, M. Alibert observes, for such assertion. Our author considers the real or *primary* seat of tinea to be in the *rete mucosum;* but that, when the disease has continued long, the different structures of the skin and subjacent cellular membrane become successively invaded.

Of M. Alibert's *post mortem and chemical* investigations, it is not our intention to take any notice, as they do not appear to lead to practical or satisfactory results.

4. *Therapeutics.* M. Alibert justly observes, that the greater the number of remedies we have for a disease, the less power we have over it. Ambrose Paré recommended surgeons not to attempt the removal of tinea—probably from being convinced that it was one of those diseases which it is "dangerous to cure." M. Alibert, without subscribing entirely to Paré's advice, is nevertheless satisfied that a too sudden removal of tinea is not without serious inconvenience, not to say danger, to many of the individuals affected with it. Thus he has lately attended a young girl, 14 years of age, who was seized with a most severe stomach complaint, and uterine discharge, on having this eruption too precipitately removed. A woman was lately sent to the Saint Louis, who lost her sight almost immediately after a strong repellent application to tinea favosa. He has seen white swellings, tabes mesenterica, phthisis, fatal diarrhœa, and various other dangerous diseases quickly developed, after an injudicious and premature cure of tinea.

At the same time, our author properly observes that the irritation which tinea produces, together with the injury which it may occasion to the skin and subjacent parts, if long continued, renders it improper for us to leave the removal of the disease entirely to the efforts of Nature, though she is generally victorious in the end.

The intimate sympathy which exists between the skin and the interior organs, and the glandular or lymphatic disorders which so frequently shew themselves during tinea, demonstrate, our author observes, that the treatment of this disease should not be confined to mere local means. We see, in this complaint, an afflux, or, as it has been termed, a "determination," to the head and its integuments:—should we then dream of checking the cutaneous manifestation, without previously, or cotemporaneously changing the balance, or rather derangement of balance in the circulation, which occasioned it? Is it not proper to encourage other evacuations which may counterbalance the effects of checking the discharge from tinea? Facts seen every day at Saint Louis illustrate these questions. It is there constantly remarked that those children who are subject to nasal hæmorrhages, or to a large discharge of fetid urine, are very little subject to tinea; or, if affected thereby, that they are very easily cured. Hippocrates, indeed, and his disciples, inculcated constitutional treatment in tinea, and endeavoured to operate a derivation by purgatives and diuretics. He also advised a strict regimen. These are all found serviceable by practitioners of the present day, and greatly assist the local means of cure.

As to topical applications, these have been multiplied almost *ad infinitum.* Every one knows the celebrated plaster in use ever since the days of Ambrose Paré, and into the composition of which, helebore, orpiment, litharge, vitriol, alum, quick-lime, mercury, and a great number of vegetable acrids or narcotics entered! The modern pitch-cap for deracinating the hair, still employed in most of the hospitals and by regular practitioners, is little less barbarous, M. Alibert thinks, than the monstrous composition abovementioned. Yet this plan has been pursued in Saint Louis (where the greatest number of tinea that can be seen in any part of Europe are collected) for very many years, the results of which are impartially stated by M. Alibert in the following words.

> " $1^{mo.}$ The space of six months was the shortest period of cure, on this plan; and the number cured thus was the smallest of any. $2^{ndo.}$ A considerable number required eleven or twelve months of this process. $3^{tio.}$ In the course of two years a great number were cured. $4^{to.}$ Three years were generally required for curing those in whom the disease was obstinate. $5^{to.}$ A few resisted the process even after the last-mentioned period. $6^{to.}$ The cure was not always permanent, there being several relapses requiring new process. $7^{mo.}$ Some children experienced severe diseases after the cure by the cap, and remained languishing and cachectic." 437.

M. Alibert condemns also the process of extirpating the hairs, one by one, with pincers, as "less effectual and still more barbarous than the preceding." "Cette méthode n'est-elle pas plus defectueuse, pour ne pas dire plus barbare, que la precedente, par les violences repetées qu'elle fait exercer sur le cuir chevalu."

M. Alibert here enumerates and descants on the various topical remedies which have been recommended in tinea, all of which were tried in the most careful manner at the Saint Louis, but with little or no success—many of them producing manifest injury. From this denunciation, however, he excepts a remedy lately much praised in Germany, and used by himself and colleagues at Saint Louis, with considerable and unexpected benefit. This is a combination of equal parts of sulphur and charcoal incorporated with common cerate, and well rubbed on the scalp, after the hair has been shaven, and the scabs softened and removed by cataplasms and fomentations.

"Those who have visited St. Louis," says our author, "can witness, that very frequently this application has been crowned with unequivocal success. "Les temoins assez nombreux savent que fort souvent nous avons vu cette nouvelle application coronnée d'un succés incontestible."

In short, he has seen more certain and speedy cures from this than from any of the applications before enumerated. Out of thirty individuals, thirteen were cured in about four months by the daily use of this application—the rest required seven or eight months. M. Alibert thinks that this application is perfectly safe, as no inconvenience in any case has resulted from its employment. He has lately succeeded in curing a most obstinate case of tinea favosa by this process, which had resisted every other means from infancy to the age of eleven years. He thinks the proportion of sulphur ought, in general, to be greater than that of the charcoal.

In order, however, to guard the constitution against the consequences of suppressing the discharge from tinea, and removing an irritation of long standing from a cutaneous surface, M. Alibert thinks that the prudent and enlightened physician will establish some other drain, as by an issue or seton—or at least take blood occasionally from the system, as an equivalent.

In aid of the local means of cure, also, internal and general means should be employed. The alimentary canal should be particularly attended to; and the warm bath, especially in those cases where other parts of the surface, besides the scalp, are affected, becomes a powerful auxiliary.

Our author observes that there are many cases of tinea, especially where the disease has not committed great ravages on the integuments, which give way to cleanliness alone; but that there are many others, where there is vice of constitution, and where the disease will not be subdued till this is corrected. The following observation of this experienced physician may be worthy the attention of some of our precipitate practitioners, who ridicule the connexion of local, and particularly cutaneous affections, with constitutional disorder.

"May I be permitted one final reflection which may account for the diversity of results which men experience in the treatment of the complaint in question. Cutaneous diseases, and of course tinea, have, like other diseases, their periods of access, increase, and decline. Practitioners are astonished that they do not succeed—while it is no uncommon thing to see them administering, from the beginning those remedies which can only be useful when the disease is on the decline. They are too impatient to follow the footsteps or indications of Nature, when she appears slow in her march, and life is short in its duration."

M. Alibert has made numerous comparative trials with all the different means, active and simple; and he found none so good and so safe as the sulphureous application above-mentioned, combined with warm bathing, cleanliness, and attention to the general health. This treatment, he affirms, is applicable to all the different species of tinea. Where the disease is very inveterate or of long standing, and where the vital properties of the integuments must be changed, he recommends, as a depilatory, an ointment, the basis of which is potassa and carbonate of lime. In the course of a few days, this application causes those hairs which grow on the diseased part to fall off, after which the scalp whitens, the itching subsides, and the cure proceeds, when aided by proper internal means.

Upon the whole, while we consider M. Alibert and our continental brethren as too much afraid of curing or repelling cutaneons eruptions, we are convinced that the practitioners of this country are rather too prone to run into a contrary extreme, and attack affections of the surface with as little ceremony as if the skin was a kind of covering quite unconnected with the rest of the constitution. We have seen so many injurious consequences result from the sudden suppression of long established discharges, and the precipitate removal of local irritations, that we cannot too earnestly exhort our junior brethren to keep their attention turned to this subject. We would recommend to their consideration the many important remarks which Dr. Parry has made on this subject in his elements of pathology, a work too little studied in the present day. It is our intention, ere long, to present to our readers a very comprehensive view of that experienced physician's work, which may perchance convince them that they are allowing a mine of great value to remain unworked, though close to the surface.

